# Rationale and clinical benefits of an intensive long-term pulmonary rehabilitation program after oesophagectomy: preliminary report

**DOI:** 10.1186/2049-6958-7-21

**Published:** 2012-07-28

**Authors:** Filippo Lococo, Alfredo Cesario, Silvia Sterzi, Giovanni Magrone, Valentina Dall’Armi, Francesca Mattei, Francesco Varone, Venanzio Porziella, Stefano Margaritora, Pierluigi Granone

**Affiliations:** 1 Department of General Thoracic Surgery, Catholic University of the Sacred Heart, Largo F. Vito 1, Rome, Italy; 2 Unit of Clinical and Molecular Epidemiology, IRCCS San Raffaele Pisana, Rome, Italy; 3 Department of Physical Rehabilitation Medicine, Campus Bio-Medico, Rome, Italy; 4 Department of Pulmonary Medicine, Catholic University of the Sacred Heart, Largo F. Vito 1, Rome, Italy

**Keywords:** Chest physical therapy, Oesophagectomy, Rehabilitation, Surgery

## Abstract

Patients who undergo oesophagectomy for oesophagealcancer (OC) usually have an overall poor prognosis and, still more preoccupying, an unsatisfactory quality of life (QoL). Considering that, as already noted, post-operative pulmonary function has a strong correlation with the long-term outcome and QoL after surgery, we have assumed and speculated on the clinical benefits of an intensive long-term pulmonary post-operative rehabilitation program in this particular subset of patients.

Herein, we report the preliminary results of a comparative retrospective analysis in a series of 58 patients who underwent radical oesophagectomy and post-operative chest physical therapy (CPT) under two different protocols, from October 2006 to January 2011.

Finally, we discuss on the time-trend analysis of pulmonary function and the potential role of post-operative pulmonary rehabilitation.

## Background

In the last decades, quality of life (QoL) assessment is increasingly being used in clinical cancer research as an important outcome for assessing treatment effects [1]. In addition, recent attention has been directed toward the possibility of employing individual QoL assessments in daily clinical practice [2]. Both efforts are aimed at factoring QoL considerations explicitly into the medical decision-making process. Given that the patient is the most appropriate source of information on his QoL, such assessments are primarily derived from the patients themselves.

In this setting, we would like to discuss and speculate on the rehabilitation strategy in oesophageal cancer treatment. Recently, Verschuur and colleagues [3] reported their results on patients’ physical fitness and the problems experienced, either physically or psychosocially, after oesophageal resection. Evidence has also been published suggesting that the impact of post-operative rehabilitation in patients undergoing oesophagectomy is beneficial both in terms of long-term outcome [4] and QoL [5]. The conclusions of the analysis of a large series by Djärv are clear: “patients who do not recover physical function, pain, and fatigue scores within 6 months after potentially curative treatment for oesophageal cancer are at significant increased risk of shorter survival” [6], implying that the management of post-operative complications and the recovery of physical function after surgery are pivotal. Inspired by these considerations, in October 2006 we planned an experimental clinical trial on intensive long-term post-operative rehabilitation after oesophagectomy in our institution.

## Methods

We retrospectively analyzed the data of 58 patients with oesophageal cancer (OC), consecutively observed and surgically treated in our department from October 2006 to January 2011, who received chest physical therapy (CPT) under two different protocols. Of these, 50 (Group A), treated from October 2006 to June 2010, underwent standard rehabilitative procedures (SR) and 8 (Group B), treated from June 2010 to January 2011, followed a new multimodal rehabilitation regimen based on intensive rehabilitative procedures (IR).

The SR consisted of coughing instruction, mainly to improve ventilation and promote expectoration. At the same time, patients were instructed to perform deep breathing and abdominal breathing as respiration training. General exercise therapy and a specific training session for the inspiratory muscles were also used to achieve early mobilization.

Regarding the IR, we adopted a multidisciplinary strategy along the lines of the protocol used for surgical Non Small Cell Lung Cancer (NSCLC) patients as described by Cesario et al. [7].

The Rehabilitation Team consisted of a chest physician director, physical therapists, nurses, a psychologist and a dietician. Subjects participated in 5 daily sessions each week, for a total of 4 weeks. The program included: (a) a supervised symptom-limited incremental exercise (cyclo-ergometer test); (b) abdominal muscle activities, inspiratory resistive sessions, treadmill, upper and lower extremities training and full arm circling; c) educational sessions, conducted twice weekly, covering such topics as pulmonary physiopathology, pharmacology of patients’ medications, dietary counselling, relaxation and stress management techniques, energy conservation principles, and breathing retraining.

Pulmonary function was evaluated before surgery (T_0_) and 1 month after discharge (T_2_) in both groups. In Group A, the evaluation was performed after surgery and before IR (T_1_). In addition, before and after IR all patients underwent specific measurements to evaluate the clinical benefit of the rehabilitation course: exercise endurance was evaluated with a 6-min walking distance (6MWD) test using the modified 6MWD protocol [8]; perceived breathlessness/dyspnoea and leg fatigue were evaluated using the modified Borg scale [9]; perceived pain was estimated by a visual analogue scale (VAS) score [10]; general physical performance was tested by the Barthel score [11]. In addition, the multifactorial index, providing information on body mass, airflow obstruction, dyspnoea and exercise index (BODE index [12]), was also measured. Finally, the “Instrumental Activities of Daily Living” (IADL) score [13] was routinely administered before and after IR to check the general status of the patients and their ability to recover a “normal daily life”.

Tests and treatment were performed as part of the clinical routine and were in accordance with the World Medical Association declaration of Helsinki [14]. Finally, an informed medical consent was also obtained from all patients enrolled in the present study.

## Results and discussion

The main demographic and clinical characteristics of the study group are reported in Table 1. In particular, chronic obstructive pulmonary disease (COPD) was found in 20 patients of Group A (40%) and 4 patients of Group B (50%), respectively. The two study groups were comparable for demographic, pre-operative respiratory function and surgical characteristics, as well as for the peri-operative morbidity (42% in Group A and 37.5% in Group B; p = ns) and for pulmonary complications (25% in Group A and 26% in Group B; p = ns).

**Table 1:** Clinical and demographic parameters of the study population

**Variables**	**Non Rehabilitated Group (n = 50)**		**Rehabilitated Group (n = 8)**		**P-value**
	**n (%)**		**n (%)**		
Sex, F		16 (32%)		2 (25%)		1.000
NAD	5 (10%)		1 (13%)		1.000	
Smoker	13 (26%)		3 (38%)		0.672	
Ex smoker	16 (32%)		3 (38%)		1.000	
Comorbility_pneumo	20 (40%)		4 (50%)		0.706	
Comorbility_other	18 (36%)		1 (13%)		0.252	
Operation type: Thoracotomy	16 (32%)		3 (38%)		1.000	
Transhiatal	34 (68%)		5 (63%)			
Early_complications	21 (42%)		3 (38%)		1.000	
Early_complications_pneumo	13 (26%)		2 (25%)		1.000	
Late_complications	9 (18%)		3 (38%)		0.342	
Late_complications_pneumo	2 (4%)		1 (13%)		0.365	
CT	14 (28%)		2 (25%)		1.000	
RT	22 (44%)		4 (50%)		1.000	
	**mean ± sd**	**median**	**mean ± sd**	**median**	**P-value**	
Age	67.46 ± 0.53	69.00	70.00 ± 5.45	69.5	0.5089	
PY	14.92 ± 5.30	10.00	20.38 ± 5.71	24.00	0.3418	
BMI	23.25 ± 1.75	23.00	22.64 ± 0.94	22.30	0.3356	
Rehabilitation days	n.a.	n.a.	15.13 ± 3.60	15.00	n.a.	

The results of pulmonary function tests and the time-trend analysis of these variables are illustrated in Table 2 and 3. As expected, almost all patients who underwent SR after surgery (Group A) showed a clear reduction of pulmonary function with a significant decrease in terms of FVC%, FEV_1_%, TLC% and RV% (p < 0.001 in all cases).

**Table 2:** Time-trend analysis of pulmonary function in Group A (SR: Standard Rehabilitation) and Group B (IR: Intensive Rehabilitation)

	**Variables**	**T0**		**T1**		**T2**		**P-valueT0-T1**	**P-valueT1-T2**	**P-valueT0-T2**
		**mean ± sd**	**median**	**mean ± sd**	**median**	**mean ± sd**	**median**			
SR Group(n = 50)	FVC pred%	97.44 ± 18.19	96.5	-	-	85.21 ± 20.03	86.00	-	-	<0.0001
	FEV_1_ pred%	88.38 ± 20.56	91.5	-	-	77.19 ± 21.73	80.00	-	-	<0.0001
	TLC pred%	95.32 ± 16.66	95.5	-	-	89.55 ± 14.8	88.00	-	-	<0.0001
	RV pred%	104.46 ± 28.18	100.5	-	-	98.43 ± 27.53	94.00	-	-	<0.0001
IR Group(n = 8)	FVC pred%	96.75 ± 13.61	98.00	92.38 ± 9.50	93.50	95.88 ± 8.39	96.50	0.0925	0.0136	0.1797
	FEV_1_ pred%	82.75 ± 13.76	79.50	78.50 ± 9.90	76.50	85.63 ± 8.25	81.50	0.0793	0.0136	0.5271
	TLC pred%	97.5 ± 14.27	100.50	87.25 ± 10.40	86.50	95.75 ± 12.16	96.50	0.0117	0.0117	0.1013
	RV pred%	107.00 ± 18.94	104.50	94.13 ± 9.40	91.50	102.88 ± 15.43	99.50	0.014	0.0138	0.0348
	6MWD	-	-	159.63 ± 29.34	152.50	223.63 ± 33.81	234.00	-	0.0117	-
	Borg	-	-	4.25 ± 0.71	4.00	2.50 ± 0.53	2.50	-	0.0083	-
	Barthel	-	-	67.13 ± 4.7	67.00	95.00 ± 4.60	95.50	-	0.0116	-
	Vas	-	-	5.38 ± 1.03	5.00	1.20 ± 0.64	1.15	-	0.0115	-
	Po2			67.29 ± 4.51	67.85	78.45 ± 5.26	77.80	-	0.0116	
	IADL Score			2,7 ± 0.43	3.00	4.2 ± 0.79	5.00	-	0.081	

**Table 3:** Comparative long-term results of pulmonary function between Group A (SR: Standard Rehabilitation) and Group B (IR: Intensive Rehabilitation)

**Group**	**Variables**	**Thoracotomy**			**Transhiatal**			**Total**			
		**n**	**mean ± sd**	**median**	**n**	**mean ± sd**	**median**	**n**	**mean ± sd**	**median**	**P-value**
IR	Delta T0-T2FVC pred. %	3	−1.71 ± 1.18	−1.16	5	−1.11 ± 12.80	−1.15	8	−0.05 ± 9.81	−1.16	0.001
SR		15	−20.68 ± 11.80	−21.28	32	−8.53 ± 5.77	−6.42	50	−12.40 ± 9.88	−9.32	
IR	Delta T0-T2FEV1 pred. %	3	−2.14 ± 5.42	−3.74	5	−9.04 ± 12.44	−3.85	8	−4.83 ± 11.41	−1.92	0.0001
SR		15	−21.28 ± 11.32	−22.22	32	−8.33 ± 6.06	- 7.69	50	−12.46 ± 10.05	−8.62	
IR	Delta T0-T2TLC pred. %	3	−1.56 ± 1.43	−1.92	5	−1.48 ± 3.47	−3.00	8	−1.52 ± 2.73	−2.36	0.0395
SR		15	−7.23 ± 6.84	−7.07	32	−4.67 ± 4.32	−5.16	50	−5.49 ± 5.32	−5.19	
IR	Delta T0-T2RV pred. %	3	−3.03 ± 2.68	−4.00	5	−3.70 ± 3.51	−5.50	8	−3.44 ± 3.03	−4.54	0.8109
SR		15	−6.82 ± 6.33	−3.90	32	−4.11 ± 5.36	−2.54	50	−4.98 ± 5.76	−3.64	

On the other hand, a clinical benefit during the course of the IR in Group B (ΔT_1_-T_2_) was found in terms of pO_2_ [MeanΔ = 8.9 days; p = 0.0116], Borg-scale [MeanΔ = 1.5; p = 0.0083], Barthel Test [MeanΔ = 28.8; p = 0.0116], 6MWD [MeanΔ = 70.4; p = 0.0117] and VAS-Score [MeanΔ = 4.18; p = 0.0115]. An improvement was also found in pulmonary function, in terms of FVC%, FEV_1_%, TLC% (p = 0.0136, p = 0.0136, p = 0.0117, respectively).

Therefore, the pulmonary function values after rehabilitation (T_2_) were substantially similar when compared with pre-operative assessment (T_0_) (Table 1).

On the contrary, patients in Group A showed an incomplete functional recovery (ΔT_0_-T_2_) with the pulmonary function strongly decreased [FVC% (p < 0.0001.), FEV_1_% (p < 0.0001), TLC% (p < 0.0001)]. In fact, comparing the variation of pulmonary function values (ΔT_0_-T_2_) in the 2 groups, the data confirm that patients who underwent IR had a significantly lesser decrease in lung function (Table 1). This functional recovery [FVC% p = 0.001; FEV_1_% p = 0.0001; TLC% p = 0.0395 ] is even more important if we consider the strong correlation with the long-term outcome and QoL of these patients, as already noted [15]. Finally, as reported in Table 2, the results of the IADL, measured before and after IR, demonstrated a slightly significant improvement (p = 0.081) in daily living activities.

Patients who undergo oesophagectomy for OC usually have an overall poor prognosis and, even more so, an unsatisfactory QoL. In this setting, Djärv et al. [5] recently investigated the possible association between baseline Health-Related Quality of Life (HRQL) and survival, and also between changes in HRQL, before and after treatment, and survival in this particular subset of patients. As previously cited, the authors concluded that the risk of dying in patients with dyspnea is significatly higher when physical function, pain, and fatigue do not recover by 6 months from curative treatment*.*

Therefore, given that QoL is clearly associated with the short-time outcome of these patients, the achievement of a good physical function after surgery represents an enduring challenge for the multidisciplinary team dealing with the management of this disease. Generally, one of the most common pulmonary modifications related to this kind of surgery consists of a moderate-to-strong post-operative change in lung function (predominantly restrictive ventilator pattern) with decreases in vital capacity (VC) and functional residual capacity (FRC). The decrease in FRC impairs ventilation/perfusion matching and sometimes results in arterial hypoxemia [16]. Several pathophysiological mechanisms could be considered as a cause of this dysfunction. Firstly, there is an inadequate pain control post-thoracotomy; in fact, a postero-lateral thoracotomy is among the most painful of surgical incisions because major muscles, including the nerves, are divided, and the ribs are distracted and partly or wholly removed. The decrease in respiratory function can last for some weeks, and this is amplified in patients with respiratory disease [17]. Moreover, thoracic incisions strongly affect the integrity of the respiratory muscles, resulting in pulmonary dysfunction and, consequently, in a change in the quality of breathing. In this context, considering the association between the post-operative pulmonary function and the post-operative QoL, as suggested by several authors [17,18], we hypothesized that the recovery of a normal lung capacity after surgery (as soon as possible, and in the best possible way) represents a factor of great importance for the overall strategy of care of the OC.

### Strengths and limitations

This paper has the usual limitations associated with retrospective mono-centric studies: the long duration of patient recruitment and the limited number of patients. Given that this is a preliminary report of a retrospective comparative analysis of two different “strategies of care” in the post-operative management of patients who underwent oesophagectomy, the number of patients in the two groups is disparate: this represents a further limitation (“intrinsic bias”) that readers should clearly keep in mind.

Moreover, we did not perform a direct evaluation of the QoL score of the patients because no specific and validated questionnaires such as EORTC QLQ-C30 [19] were administered during the study period, since the analysis focused mainly on post-operative pulmonary functional values. In any case, the IADL score was administered in all patients who underwent the rehabilitation program and it can be considered as a surrogate method (indirect evaluation) to gain some information on QoL, as already validated by previous experiences [20-22].

Despite all the limitations mentioned above, this study has the merit of highlighting the critical role of a multidisciplinary post-operative management of patients who have undergone oesophagectomy, and suggests that, in such patients, the respiratory function and exercise capacity significantly improve after an intensive outpatient long-term (4-week) pulmonary rehabilitation program. Inspired by these preliminary results, we have planned a prospective randomised controlled trial. In this ongoing trial the positive results described in this study will be verified along with a proper QoL evaluation using specific and widely accepted scoring systems.

## Conclusions

As previously suggested, post-operative rehabilitation in patients undergoing oesophagectomy is beneficial both in terms of long-term outcome [4] and QoL [5]. According to our preliminary experience, a multimodal outpatient long-term rehabilitation program could be considered as a key component of the management of this subset of patients, providing clinical benefits and a potential improvement in QoL.

However, the validation of this approach and its translation into everyday clinical practice require further investigation.

## Abbreviations

BMI, Body Mass Index; BODE, Body mass, airflow Obstruction, Dyspnoea and Exercise; COPD, Chronic Obstructive Pulmonary Disease; CPT, Chest Physical Therapy; CT, Chemotherapy; EORTC QLQ-C30, European Organization for Research and Treatment of Cancer Questionnaire for the Quality of Life composed of 30 Core questions; FEV1%, Forced Expiratory Volume in 1 second (percentage of the theoretical value); FRC, Functional Residual Capacity; FVC%, Forced Vital Capacity (percentage of the theoretical value); HRQL, Health-Related Quality of Life; IADL, Instrumental Activities of Daily Living; IR, Intensive Rehabilitative procedures; NAD, Neoadjuvant Therapy; NSCLC, Non Small Cell Lung Cancer; OC, Oesophageal Cancer; pO2, Oxygen Pressure; PY, Pack/years Index; QoL, Quality of Life; RT, Radiotherapy; SR, Standard Rehabilitative procedures; T0, before surgery; T1, after surgery and before IR; T2, 1 month after the discharge; TLC%, Total Lung Capacity (percentage of the theoretical value); VAS, visual analogue scale; VC, Vital Capacity; Δ, Delta o Differenza; 6MWD, 6-min Walking Distance test.

## Competing interests

The authors declare that they have no competing interests.
